# Immediate titanium mesh cranioplasty after debridement of post-craniotomy infection

**DOI:** 10.1007/s00701-025-06509-4

**Published:** 2025-06-04

**Authors:** Micaela Uberti, Navneet Singh, Andrew J. Martin

**Affiliations:** Department of Neurosurgery, St. George’s Hospital, London, UK

**Keywords:** Titanium mesh, Surgical site infection, Bone-flap removal, Immediate cranioplasty, Delayed cranioplasty

## Abstract

**Purpose:**

For post-craniotomy surgical site infection (SSI) involving the bone, typical management involves removal of the bone flap and delayed cranioplasty. The disadvantages of delayed cranioplasty include cosmetic deformity, vulnerability of unprotected brain, skin contraction, syndrome of the trephined and the risks of further surgery. Second procedures also add to cost due to surgical time, hospital stay, and opportunity costs for patients from being away from work.

**Methods:**

We retrospectively reviewed patients who underwent post-craniotomy bone flap removal due to SSI, with immediate titanium mesh cranioplasty. The primary outcome was re-operation due to persistent infection or wound healing complications. The secondary outcome was re-operation due to unacceptable cosmetic result.

**Results:**

Nineteen patients were included between 2018 to 2024. Two patients required additional debridement and removal of the titanium mesh due to persistent infection. Another patient had the plate replaced with PEEK due to poor skin quality, wound breakdown and an unacceptable cosmetic result. Two further patients with bifrontal craniotomies had the mesh replaced for cosmetic reasons. Fourteen patients had long term resolution with no further procedure.

**Conclusion:**

Immediate TM insertion at the time of bone flap removal is an acceptable option in the management of post-craniotomy SSI. It seems the overall complication rate is comparable to delayed titanium cranioplasty, with the benefit of avoiding the risks and costs of a second operation. Cosmetic results are worse with larger defects, but these patients can still benefit from early TM placement by making operative conditions easier when the custom made, delayed cranioplasty is inserted.

## Background

The risk of surgical site infection (SSI) after craniotomy ranges from 0.5% to 11% (1–3, 4), and varies with the population and the presence of well-known factors such as irradiation, previous surgery, cerebrospinal fluid (CSF) leak, duration of surgery, emergency surgery, paranasal sinus involvement, and absence of antibiotic prophylaxis [2, 7, 13, 16].

For post-craniotomy surgical site infection (SSI) involving the bone, typical management involves debridement, removal of the bone flap, antibiotic treatment and delayed cranioplasty. There is no consensus as to the appropriate time to replace the bone flap or the material. Typically, once the wound has healed, a cranioplasty is performed using a prosthesis made of one of several synthetic materials including acrylic, polyetheretherketone (PEEK), titanium and hydroxyapatite with no clear evidence that one material is superior to the other. The disadvantages of delayed cranioplasty include cosmetic deformity, vulnerability of unprotected brain, skin contraction, syndrome of the trephined and the risks associated with further surgery. In addition, second procedures add to cost due to surgical time, hospital stay, and opportunity costs for patients from being away from work during inpatient stay and post-discharge recovery.

﻿In spine surgery, titanium spinal implants have long been used in the setting of spinal pyogenic and tuberculous osteomyelitis and discitis with significant bone destruction, progressive deformity, neurological impairment, antibiotic-resistant sepsis or recurrence of infection [5].

We regularly place an immediate titanium mesh cranioplasty (TM) at the time of wound debridement and bone flap removal. In patients with a small bone flap this is generally the definitive treatment, and for those with larger defects a mesh cranioplasty provides a safe temporary measure while preparing for the delayed insertion of a custom-made prosthesis. It also prevents adherence of the scalp to the dura allowing for easier scalps dissection at the time of re-opening. We report our experience with this method and compare it with the rate of complications occurring after delayed cranioplasty with custom-made plates.

## Methods

We retrospectively reviewed paediatric and adult patients who underwent post-craniotomy wash out and bone flap removal due to SSI, with immediate titanium mesh cranioplasty. The primary outcome was re-operation due to persistent infection or wound healing complications. The secondary outcome was re-operation due to unacceptable cosmetic result.

## Results

An immediate mesh cranioplasty is standard practice for four of thirteen consultant neurosurgeons in the department. Nineteen patients met the inclusion criteria between 2018 to 2024 and all but one patient had craniotomy for tumours (Table 1). The mean time from craniotomy to infection and wash out was 2 months. Median follow up was 2 years. Two patients required additional debridement and removal of the titanium mesh due to persistent infection. Both patients had had chemo and radiotherapy after the index craniotomy as part of the treatment for glioblastoma. Due to persistent wound healing problems, neither had further surgery to cover the bone defect once the mesh was removed. Another patient had the plate replaced with PEEK due to poor skin quality, wound breakdown and an unacceptable cosmetic result. Two further patients had the mesh replaced for cosmetic reasons and both had had large bifrontal craniotomies. In their cases placing a temporary TM immediately after removing the infected bone flap was planned as a measure to prevent skin retraction and facilitate the second surgery with the customed-made plate. Fourteen out of nineteen patients had long term resolution and did not require a further procedure.

**Table 1:** Results

Patient	Original operation	Risk factors for infection	Time to wash out	Pus in theatre?	Bug Isolated	Cosmetic result	Complications	Titanium plate removed?	Follow up time
MS	Left parasagittal MNG (WHO I)	None	5 months	Yes	None	Acceptable	None	No	5 years
KR	Right clinoidal MNG	None	1 month	Yes	None	Unacceptable	Aspiration × 2 of chronic blood collection after removal of bone flap, wound dehiscence	Yes, replaced with PEEK private op	1 year
IJ	Anterior fossa MNG	DMII, smoker	2 months	Yes	Cutibacterium acnes	Unacceptable, had to have second titanium cranioplasty	None	No, has both	3 years
BM	CPA MNG	ETV before the MNG resection	2 weeks	Yes	MSSA	Acceptable	None	No	5 months
ND	Parasagittal MNG	None	2 weeks	Yes	Citobacter koseri	Acceptable	None	No	2 and a half years
PB	Frontal MNG	Ex smoker	8 days	Yes	Enterobacter	Unacceptable, had to have second titanium cranioplasty	None	Yes, replaced with titanium a year later	3 years
NAH	Sphenous wing MNG	Smoker	3 months	Yes	Cutibacterium acnes	Acceptable	None	No	2 years
DP	Chronic SDH (ITP)	EVD post first op, CSF leak from EVD wound, 1st wash out replaced bone flap 2 months after first op but this did not work.	3 months	Yes	Cutibacterium acnes	Acceptable	None	No	1 year 8 months
AB	Frontal Glioblastoma	Finished radiotherapy1 week before infection developed	2 months	Yes	Staph epidermidis + C.acnes	Acceptable	Yes	Yes, due to infection 5 months later, grew Staph aureus. Plate not replaced	8 months
AMR	Occipital metastasis	Cancer, CSF leak, smoker	1 month	Yes	Staph aureus	Acceptable	None	No	No FU available
MP	Glioblastoma	Radiotherapy, smoker	11 months	Yes	Multibacterial	Acceptable	Yes	Yes, 2 months later due to infection, bone flap not replaced	2 years, 7 months
JW	Tectal plate PA	None	2 months	Yes	Staph aureus	Acceptable	None	No	1 year
AA	Parietal sarcoma	Smoker	1 month	Yes	MSSA	Acceptable	None	No	11 months
PF	Parietal Glioblastoma	DMII	1 month	Yes	Klebsiella	Acceptable	None	No	5 months
SP	Frontal MNG	None	2 months	Yes	None	Acceptable	None	No	5 months
LB	Cerebellar PA	CSF leak from wound	1 month	Yes	Staph	Acceptable	None	No	5 months

## Discussion

In most departments, delayed cranioplasties are the standard of care after surgical site infection and removal of bone flaps. There are, however, complications that can result in the period waiting to have the new bone flap inserted. The unprotected brain is vulnerable to trauma, the syndrome of the trephined, seizures, hydrocephalus, CSF leak, and further infection, and scalp can both adhere to the dura and contract into the bony defect making any future surgery more difficult. There is also a negative cosmetic appearance and psychological stigma during this time and the added cost of a further surgery. Alternative methods to avoid this morbid procedure have been sought [1, 2, 4, 5, 8](Table 2). ﻿﻿These solutions have had acceptable outcomes, but extensive peri and postoperative effort and have fallen out of favour amongst surgeons.

**Table 2:** Attempted methods to salvage an infected bone flap in patients with SSI

Author	Year	Number of patients	Method	Result
Koslow	1979	5	Immediate stainless steel mesh + IV antibiotics post-op	5 patients had no recurrent infection
Larsson	2002	15	Hyperbaric oxygen treatment and intravenous antibiotics	12 patients had no recurrent infection
Bruce	2003	13	Scrubbing of the infected bone flap and soaking in povidine-iodine solution + IV antibiotics	11 patients had no recurrent infection
Auguste	2006	12	Suction-irrigation system used to salvage the infected bone flap	11 patients had no recurrent infection
Delgado-Lopez	2009	5	Scrubbing of the infected bone flap + autocalving or further soaking in povidone-iodine solution or chlorhexidine + 2 drains in the subgaleal or epidural space for noncontinuous irrigation + normal antbiotic treatment	5 patients had no recurrent infection

Immediate titanium mesh is a reasonable choice for cranioplasty based on strength, durability, cost, low potential for infection, suitability for postoperative imaging, malleability and good cosmesis [3, 13]. Four papers have been published advocating for immediate TM after bone flap removal, with good infection resolution [5, 7, 11, 16], (Table 3). The main risk factors for infection were preoperative radiation and postoperative CSF leak. Talwar in 2020 [13] published their literature review of 40 patients who underwent immediate cranioplasty after bone flap debridement. They report a 5% rate of postoperative infection, a 12.5% rate of unplanned return to the operating room, 7.5% rate of CSF fistula or leak, a 2.5% rate of hematoma, and a 2.5% rate of mortality within the immediate post- op period. In their review, the overall rate of infection in delayed post-infectious cranioplasties (11.5%) was higher than the 5% rate in the immediate TM group, although that difference wasn’t statistically significant.

**Table 3:** Case series looking at immediate TM after infected bone flap removal

Author	Year	Patients	Indication for craniotomy	Median follow up	Resolution without additional surgery	Complications (N of Patients)
Kshettry	2012	12	Brain tumour	14 months	10 patients	2: wound healing problems 1: persistent infection, successful new TM 1: persistent infection but opted for hospice care
Wind	2013	2	Brain tumour	3 years	2 patients	None
Ehrlich	2016	24	Brain tumour, vascular, trauma	4.6 months	22 patients	3: post-op CSF fistula 1: Persistent HCF and CSF fistula, had VPS inserted and TM was left insitu. No infection 1: Persistent CSF fistula after a chronic SDH. Dura was re-closed and TM removed. No further complications
Potter	2023	48	Tumours, vascular, trauma	20.5 weeks	36 patients	6: re-operation for wound healing issues 6: re-operation due to infection

### Comparison with delayed titanium cranioplasty

There are few studies focusing on delayed titanium cranioplasty specifically after surgical site infection [6, 9, 10, 14, 15]. Not all of them mentioned how many plates had to be removed. Our own centre (Mukherjee et al. [10]) published a review in 2014 of 174 patients with delayed titanium cranioplasty. The overall complication rate was 26.4% (46/174), the commonest being repeat infection. ﻿ The infection rate following delayed TC in patients who originally had removal of an infected bone flap following primary surgery was 20% (3/15). ﻿Infection accounted for 69% of cases of plate removal (12/ 174). The second most common complication was seizures (8%). ﻿For the 18 cases of cranioplasty removal, the median time from insertion to removal was 55 days (range 6–487). For infected cranioplasties (n = 12) the median time to removal was 41.5 days (range 30–91). The one plate removal for extradural haematoma (EDH) was performed at two days, whilst plate removal for cases of poor cosmesis (with a view to re-do surgery) was at a median of 87 days (n = 5). Patients with a skull defect larger than 100 cm2 or bifrontal defects had the highest complication and plate removal rate, and longest postoperative hospital stay. In this series, the size of defect and traumatic aetiology were risk factors for complications. Their results also suggested that the timing of cranioplasty could be important with late (> 12 months) TC associated with a higher rate of complications. ﻿﻿Interestingly, in Scott Hill’s series in 2012 [6], there was no association between interval since primary operation and complications. Although statistically not significant, a longer interval appeared to be related to post-operative infection and the need for reoperation or removal of cranioplasty. Matsuno [9] in 2006 also looked at the complications using a range of materials including autogenous bone (54 patients), polymethylmethacrylate (PMMA) (55 patients), custom-made PMMA (3 patients), and custom-made ceramics (17 patients). They concluded ﻿that autoclaved and autogenous bone grafts and PMMA had a significantly higher rate of graft infection. Titanium mesh had the lowest rate of graft infection (Table 4).

**Table 4:** Case series looking at delayed custom-made titanium cranioplasty

Author	Year	Patients	Follow up	Infection rate	Other complications	Comments
Matsuno	2006	77	10 years	2.60%		Patients who had the delayed TC for infection did not develop TM infection afterwards
Wiggins	2013	127	21 months	16%		TC for a larger defect (bifrontal or hemicranial) was associated with a higher complication rate)
Scott Hill	2012	95	42 months	13%	CSF leak (1), EDH (4), Moving plate (1), Subgaleal collections requiring further surgery (5)	Overall removal rate: 8.4%
Mukherjee	2014	174	7 years	8.60%	Seizures: 8%	Plate removal rate: 10.3% (18 patients, 12 due to infection) Patients with larger skull defects had the highest complication rate

Sadhwani [12] in 2023 looked at the infection rates following immediate and delayed cranioplasty for post-craniotomy SSIs. This covered all plate materials including titanium mesh. In a meta-analysis up to the year 2021, the mean age of the patients in the studies ranged from 3 months to 82 years. The pooled proportion of treatment failure in the immediate cranioplasty group (12 studies) was 10.4%. ﻿ ﻿The pooled proportion of treatment failure in the immediate autologous cranioplasty group (7 studies) was 12%. The pooled proportion of treatment failure in the immediate synthetic cranioplasty group (5 studies) was 8%. However, the difference between these 2 subgroups was not statistically significant. The pooled proportion of treatment failure in the delayed cranioplasty group, which included 6 studies, was 16.1%. ﻿Based on their analysis, they concluded that immediate cranioplasty be considered in patients with post-craniotomy SSI. It should even be considered in patients with risk factors for poor wound healing, such as prior craniotomy, steroid use, and radiation therapy. ﻿However, they suggested that frank purulence was a contraindication for immediate cranioplasty, and cranioplasty should be delayed in such cases. In addition, they remarked on an adequate dural closure, as in a few of the studies included CSF leaks were commonly seen and were the cause of the return to the operating room.

### Cosmetic results

In our series, two patients had revision surgery purely due to unsatisfactory cosmetic results. Both had large bifrontal craniotomies. There are few other studies looking at cosmesis of the final cranioplasty result. Ehrlich [5] used a self-designated questionnaire to evaluate the patients´ satisfaction with main focus on the cosmetic result. 20 patients out of 24 (83%) were satisfied with the cosmetic result of the surgery (with immediate TM after bone flap removal). Cabraja [3] in 2009 evaluated pain and cosmesis in ﻿26 patients who underwent delayed cranioplasty with custom made titanium implants. All patients had large defects (mean diameter 112 mm) and 14 had had the bone flap removed originally due to infection. ﻿ The follow up was 6–12 years (mean 8.1 years) after insertion of the implants and 88% were satisfied with the cosmetic result. ﻿None of the implants had to be removed, and they did not report any infection. ﻿ We believe that even large defects can benefit from immediate TM cranioplasty. It not only provides temporary cosmesis, but importantly, also avoids scalp contraction and adherence if a more definitive surgery is planned. However, it can also be the definitive treatment as well.

### Technical note

To achieve good cosmetic results specifically with titanium mesh, there are some important considerations. Wound tension can be relieved with galeal undermining, and/or the incision must be lengthened. To mimic the bone contour, we use anvil forceps and plate scissors, with careful trimming and flattening of the cut mesh edges. Titanium is a strong material, especially with small convex plates, so multiple screw fixation points are required.

#### Limits of the study

We acknowledge limitations to our study. It is a retrospective case series without a comparable control group, which limits assessment of comparative effectiveness with other methods. The study population is small and median follow up relatively short.

## Conclusion

Immediate titanium mesh insertion at the time of debridement and bone flap removal is an acceptable option in the management of post-craniotomy surgical site infection. The reported literature is based on small case series only, which limits the ability of comparing data sets. However, it seems the overall complication rate is comparable to delayed titanium cranioplasty, with the benefit of avoiding the risks and costs of a second operation. Cosmetic results are perhaps worse with larger defects, but these patients can still benefit from early TM placement by making operative conditions easier when the custom made, delayed cranioplasty is inserted. We recommend an immediate titanium mesh cranioplasty is considered in all cases where an infected bone flap is removed.

## Data Availability

No datasets were generated or analysed during the current study.
